# Dysregulation of Protein Kinase CaMKI Leads to Autism-Related Phenotypes in Synaptic Connectivity, Sleep, Sociality, and Aging-Dependent Degeneration in *Drosophila*

**DOI:** 10.3390/biology14091228

**Published:** 2025-09-09

**Authors:** Claudia Gualtieri, Zachary M. Smith, Abby Cruz, Ziam Khan, Conor Jenkins, Ketu Mishra-Gorur, Fernando J. Vonhoff

**Affiliations:** 1 Department of Biological Sciences, University of Maryland, Baltimore County, Baltimore, MD 21250, USA; 2 Department of Chemistry and Biochemistry, University of Maryland, College Park, MD 20742, USA; 3 Department of Neurosurgery, Yale School of Medicine, New Haven, CT 06510, USA

**Keywords:** autism spectrum disorder, synaptic pruning, circadian rhythm, neurodevelopment, neurodegenerative, proteomics, locomotion, activity-dependent, chemorepulsion, invertebrate

## Abstract

Autism spectrum disorder (ASD) encompasses a range of conditions, primarily marked by deficits in social behaviors, along with several comorbidities such as sleep abnormalities and motor dysfunction. Recent studies have identified genetic risk factors associated with ASD, including the *CAMK4* (calcium/calmodulin-dependent protein kinase 4). However, the molecular mechanisms linking *CAMK4* dysregulation and ASD-associated phenotypes remain poorly understood. Here, we used *Drosophila melanogaster* as a model system to investigate ASD-associated phenotypes in flies with dysregulated *CaMKI*, the fly homolog of mammalian *CAMK4*. We show that *CaMKI* manipulations affect sleep, circadian rhythmicity, and social behavior. Consistent with the higher prevalence of dementia observed in autistic patients, we also observed a significantly enhanced behavioral decline in motor performance and dendritic degeneration in flies expressing RNAi-based *CaMKI* knockdown in flight motoneurons, suggesting a link between developmental and degenerative processes. As aberrant synaptic pruning is hypothesized to underlie the synaptic phenotypes observed in brains of autistic patients, we examined synaptic phenotypes following *CaMKI* manipulations using the larval neuromuscular junction (NMJ) and observed miswiring phenotypes suggesting aberrant synaptic refinement. We performed shotgun mass-spectrometry proteomics and identified various molecular candidates, particularly molecules involved in cytoskeleton regulation and chemorepulsion, likely to regulate the phenotypes described here. Thus, our results suggest that *CaMKI* plays a role in developmental processes and influences aging-dependent degenerative processes, possibly providing mechanistic insight into the genetic basis of ASD etiology and the development of effective treatments.

## Simple Summary

This study explores the role of the gene *CaMKI* (calcium/calmodulin-dependent protein kinase I) in fruit flies, which is similar to the human gene *CAMK4*. *CAMK4* has recently been linked to autism spectrum disorder (ASD), a condition that affects social behavior and is often accompanied by sleep problems and motor difficulties. While it is known that *CAMK4* is connected to ASD, the exact ways it influences the brain and behavior are still unclear. We found that reducing *CaMKI* levels in flies affected their sleep patterns, day/night activity cycles, and social interactions. Because ASD patients are also more likely to develop dementia, we tested if altering *CaMKI* would influence age-related changes. Indeed, flies with reduced *CaMKI* showed faster declines in movement ability and signs of cell damage as they aged. Another theory about ASD suggests that it may involve problems with how connections between nerve cells, called synapses, are refined during development. In our experiments, reducing *CaMKI* caused wiring errors in the fly nervous system, which supports this idea. We also analyzed the proteins present in flies with altered *CaMKI* and identified several molecules that could explain these effects. Overall, our results suggest that *CaMKI* influences both early development and age-related brain changes. This work may provide new clues about how genetic changes in *CAMK4* might contribute to ASD in humans and could help guide the development of future treatments.

## 1. Introduction

Postmortem studies reveal increased density of synapses in brains of individuals with autism spectrum disorder (ASD) [1,2,3]. These observations have led to the hypothesis that defects in synaptic pruning may contribute to ASD pathology [4,5,6,7,8]. Synaptic pruning, also called synaptic elimination or refinement, is a neuroplastic process involving the removal of ectopic synapses formed in the initial stages of neuronal development [9,10]. Studies in vertebrates and invertebrates indicate that a crucial factor regulating synaptic pruning is neuronal activity [11,12,13], which was first hypothesized by Nobel laureates Hubel and Wiesel in the 1960s [14,15]. Studies at the vertebrate retina [16,17] as well as the mammalian and *Drosophila* neuromuscular junction (NMJ) [18,19] indicate that waves of prenatal electrical activity and calcium (Ca^2+^) oscillations regulate the withdrawal of off-target synaptic contacts [13]. Ca^2+^ oscillations regulate Ca^2+^-dependent molecular factors such as kinases (e.g., CaMKII [18,19]), phosphatases (e.g., Calcineurin [19]), and Ca^2+^-dependent adenylyl cyclases (e.g., Rutabaga [20]), which in turn regulate intracellular cyclic AMP (cAMP) levels for synaptic pruning in mammalian visual neurons [21,22] and at the *Drosophila* NMJ [20]. Whereas a model to explain the refinement of synaptic connections often involves mechanisms based on Hebbian-like, spike-timing correlation between synaptic partners, where asynchronous inputs are removed [23], alternative non-Hebbian mechanisms have also been proposed that rely on activity-dependent modulation of chemorepulsion [13]. Although some molecular aspects underlying activity-dependent synaptic refinement have recently been unraveled [13,24,25], what molecules link dynamic levels of neuronal activity and the refinement of synaptic contacts during development remain to be elucidated. Whereas some anatomical differences are observed between brains of humans and animal models, including rodents and flies, a large body of the literature indicates a high level of conservation in basic organizational principles for neuronal network structure, assembly and function, ranging from neuronal and synaptic anatomy to biophysical and electrical properties [26,27,28,29,30,31]. Consistent with its contributions to advance our understanding of synaptic development, *Drosophila melanogaster* has emerged as a suitable genetic model system for investigating fundamental neuronal mechanisms associated with ASD [32,33,34,35].

*CAMK4* (calcium/calmodulin-dependent protein kinase 4) has recently been identified as a risk gene for autism spectrum disorder [36]. Evidence from various studies using whole-exome sequencing, candidate gene association, and copy number variation indicates numerous genetic variations in *CAMK4* in populations diagnosed with ASD [37,38,39,40]. Consistently, the Simons Foundation Autism Research Initiative (SFARI) gives *CAMK4* a SFARI Gene Score of two, signifying it as a strong candidate autism risk gene. Although *CAMK4* is known to encode a serine/threonine protein kinase involved in a Ca^2+^-calmodulin dependent pathway that regulates synaptic plasticity and other neuronal functions, experimental evidence linking *CAMK4* function with autism-related phenotypes remains elusive. *CAMK4* is evolutionary conserved from yeast, worms, and flies to zebrafish, rodents, and humans, including the *Drosophila* ortholog *CaMKI*. In response to social experience, *Drosophila CaMKI* is known to activate the histone-acetyl transferase *CBP*, which enhances the efficacy of Juvenile Hormone and the transcription of *fruM* in male olfactory neurons to modulate pheromone detection and courtship behavior [41,42]. However, whether dysregulation of *CaMKI* signaling leads to autism-associated phenotypes remains an unexplored question.

Here, we used *Drosophila* as a model to test *CaMKI* manipulations and their effect on autism-related phenotypes. Despite limitations of experimental animal models in resembling human neurotypical behaviors and clinical symptoms, their contribution to the understanding of the etiology and pathogenesis of human diseases, including ASD, is widely accepted considering the high degrees of evolutionary conservation of the nervous system and behaviors [43,44]. Consistently, social behavior is observed in various animal taxa ranging from insects to large mammals [44,45,46], and sleep has been well documented throughout the entire animal kingdom, ranging from the nematode worm *Caenorhabditis elegans* to elephants [47]. ASD involves a spectrum of disorders/conditions, mainly characterized by deficits in social behaviors, which can also include various comorbidities such as sleep abnormalities [48,49,50,51,52]. Therefore, we tested whether *CaMKI* manipulations had an effect on ASD-related behaviors such as sleep and sociality in adult *CaMKI* loss-of-function mutants (*CaMKI*^LOF^) as well as in animals expressing pan-neuronal *CaMKI* knockdown. Following both manipulations, we observed that the disruption of *CaMKI* function led to increased sleep levels, but *CaMKI*^LOF^ flies showed decreases in sociality and circadian rhythmicity.

Moreover, recent studies indicate a higher prevalence of dementia diagnosis in ASD patients [53,54,55,56,57]. However, whether genes that regulate neuronal development can affect neurodegeneration processes remains poorly understood. Consistent with previous studies establishing *Drosophila* as a key model for aging research [58,59], we tested the effects of *CaMKI* manipulations on aging flies and observed that flight performance declined more severely in flies expressing RNAi-knockdown of *CaMKI*. Anatomical analysis revealed that this behavioral phenotype correlated with enhanced degeneration of motoneuron dendritic architecture. These phenotypes in the motor network and locomotion performance are consistent with the high prevalence of motor dysfunction observed in ASD patients [60,61], considered a comorbidity in ASD. From our proteomic analysis, we identified the β-amyloid precursor protein binding protein 1 (APP-BP1) [62] as well as the kinase Par1 [63] as molecular candidates linking neurodevelopment and degeneration. Thus, our results suggest that *CaMKI* plays a role in developmental processes and influences aging-dependent degenerative processes.

To assess synaptic perturbations at the anatomical level, we used the larval neuromuscular junction (NMJ) as previous studies have established it as a powerful model to study various aspects of synaptic connectivity including activity-dependent synaptic refinement and plasticity [13,64,65,66,67,68,69,70,71,72,73,74]. Thus, we performed anatomical analysis of NMJ connectivity and observed an increase in the frequency of ectopic connections in *CaMKI*^LOF^ mutants as well as in animals expressing pan-neuronal *CaMKI* knockdown. Epistatic genetic interaction tests suggest that *CaMKI* may be part of a different molecular pathway than the activity-dependent regulation of *PlexB/Sema2a* chemorepulsion [13]. Therefore, to identify molecular mechanisms underlying the phenotypic alterations described here, we performed shotgun proteomics on *CaMKI*^LOF^ mutants to reveal genes that show significant changes in protein levels and phospho-peptides. We identified *CaMKI*-dependent interactions with the cAMP-phosphodiesterase Dunce and the phosphatase CanB that we have previously described to regulate synaptic refinement [19,20]. Thus, dynamic phosphorylation levels of target proteins can be hypothesized to be regulated by an interplay between CaMKI-dependent phosphorylation and CanB-dependent dephosphorylation. Interestingly, additional neuronal proteins identified in our dataset known to regulate neuronal anatomy and cytoskeleton are the small GTPase Cdc42 that regulates the actin cytoskeleton and cell polarity [75], and Futsch, a MAP1B-like protein required for dendritic and axonal development implicated in the regulation of microtubule dynamics [76]. Furthermore, we identified several proteins known to regulate repulsive axon guidance mediated by the presynaptic Plexin A (PlexA) receptor [77,78]. These include the actin regulatory oxidation–reduction enzyme MICAL-like that directly bind and disassemble actin filaments (F-actin) [79], the A kinase anchoring protein (AKAP) Nervy [80], and the phospho-binding protein 14-3-3ε [81]. Therefore, our results point to an alternative signaling pathway that involves PlexA in the regulation of synaptic connectivity.

## 2. Materials and Methods

### 2.1. Fly Stocks

Flies were kept at 25 °C in a 12/12 h light/dark incubator on standard plastic vials, using a regular cornmeal/molasses food as described before [82,83]. As the wildtype line, we used DGRP-774 (RRID:BDSC_25205) from the Drosophila Genome Reference Panel lines, which are fully sequenced inbred stocks derived from a natural population from Raleigh, NC [84]. The following fly lines were obtained from the Bloomington Drosophila Stock Center: *CaMKI*^LOF^ (RRID:BDSC_16799) [41]; elav^C155^-GAL4; UAS-Dicer (RRID:BDSC_25750); UAS-*CaMKI*-RNAi-1 (RRID:BDSC_41900) [85,86]; UAS-*CaMKI*-RNAi-2 (RRID:BDSC_35362) [41,85,86,87]. Mutant lines used in genetic interaction tests as previously described [18,19] are as follows: *CaMKII*^LOF^ (RRID:BDSC_60770), *CanA-14D*^LOF^ (RRID:BDSC_22025), *cac*^LOF^ (*cac^NT27^*), *nap^ts^*; *TipE* (Na(v)1 voltage-gated Na channel mutants *mle^nap-ts^; tipE*; [88]), *Sema2a*^LOF^ (*Sema2a^B65^*), w^1118^. The C380-GAL4, UAS-mCD8-GFP; Cha-GAL80 expresses GAL4 predominately in adult motoneurons as previously described [89]. The *CaMKI*^LOF^ mutant line was created by a single P-element insertion in the CaMKI gene as part of the Gene Disruption Project [90], and the loss-of-function (LOF) nature of this mutation was confirmed in our proteomic analysis as described below. We preferred the use of *CaMKI*^LOF^ mutants for most of our experiments as we expected stronger phenotypes than RNAi-based knockdown manipulations. The only exception involved the quantification of flight performance decline and motoneuron dendritic degeneration that was assessed only in RNAi flies due to the possibility to knock down *CaMKI* exclusively in motoneurons and estimate *CaMKI*-dependent effects on the motor network in a more precise manner. Generally, RNAi manipulations were primarily used to provide evidence of the specificity of the *CaMKI*-dependent effects in behavioral (sleep) and anatomical (NMJ miswiring) assays.

### 2.2. CaMK Homologue Visualization

Visualization of CaMK domains and orthology was performed using IBS2.0 web server [91]. The protein domain map of *Drosophila* CaMKI (Q7JMV3) and Human CAMK4 (Q16566) are shown. Identity of domains calculated from alignment of CaMKI (NP_524622.1) and CAMK4 (NP_001310303.1) on DIPOT (v9) [92].

### 2.3. Sleep and Circadian Rhythm Experiments

Sleep timing and sleep bouts were assessed in <10-day old male flies using the TriKinetics Drosophila Activity Monitoring System (DAMS; TriKinetics, Waltham, MA, USA). Data were collected with DAMS every 1 min for 3 or 4 consecutive days in 12-h light/12-h dark (LD) followed by 5 or 6 days in constant darkness (DD). Flies, initially kept at a standard 12:12 light/dark cycle, exhibit bimodal behavior. Zeitgeber is the German word used to indicate a rhythmically occurring natural phenomenon, onset/offset of light in this study, which acts as a cue in the regulation of the body’s circadian rhythms. In LD conditions, Zeitgeber time (ZT) is used to indicate the time in 24 h cycle, with ZT0 being the “lights on” time, ZT12 the “lights off” time, and ZT24 the “lights on” time again. In the absence of external cues though, such as in constant dark conditions, subjective day is the portion of that 24 h cycle that the organism perceives as daytime [93]. Given that the DD condition is followed by the standard 12:12 LD condition, the flies are “primed” to perceive ZT0-ZT12 as their subjective day and ZT12-ZT24 as their subjective night in the absence of light cues. Therefore, subjective day or night is defined as the interval in DD that corresponds to the day or night phase under prior LD cycling conditions. Indeed, in the absence of external light cues, time is not defined in reference to a Zeitgeber but in reference to the internal circadian rhythmicity; therefore, “Zeitgeber Time” (ZT) is substituted by “Circadian Time” (CT). Data were analyzed using Visualization and ANalysis of timE SerieS dAta—Drosophila Activity Monitors (VANESSA-DAM, v1.0.3) [94] to generate actograms and sleep/circadian metrics. Specifically, VANESSA-DAM-CRA was used for circadian rhythm analysis, and VANESSA-DAM-SA was used for sleep analysis. Sleep was defined as five or more consecutive minutes of inactivity [95,96,97]. Daytime sleep was classified as sleep occurring during the lights-on phase (Zeitgeber Time (ZT) 0–ZT12), while nighttime sleep was defined as sleep occurring during the lights-off phase (ZT12–ZT0). Sleep parameters, including total nighttime sleep and the number and duration of sleep bouts, were averaged for each genotype. The power–significance threshold value, often referred to as the relative power, indicates how much stronger the observed rhythmic signal is compared to the background noise level, and it is used as an indicator of rhythm robustness. Using the VANESSA-DAM software, under chi-square analysis of periodograms, for a given *p*-value (*p* < 0.05), we calculated a power threshold based on number of data points, variance, and distribution of power across the tested periods. Then, the significance threshold line was plotted on the periodogram, and any fly with a power above this line is rhythmic. The robustness of the circadian rhythm was then determined by computing the difference between the peak chi-squared value and the value expected by chance. The rhythmicity index (RI), another measure of robustness of circadian rhythmicity, was also calculated using autocorrelation-based periodograms [94], and a frequency distribution was obtained (RIs were binned according to the following criteria: RI < 0.1-arrhythmic, 0.1 < RI < 0.3-weakly rhythmic, and RI > 0.3-rhythmic). The Fisher exact test applied to the RI frequency distributions also confirmed a significant difference in rhythmicity between the controls and *CaMKI*^LOF^ mutants (statistical analysis not shown). In order to examine possible locomotion defects, we calculated the activity index (or waking activity index), which is a measure of activity counts per waking minute. Activity index calculations and graphs were directly obtained from the VANESSA software for DAM data analysis.

### 2.4. Anatomical Approaches and Flight Performance

Adult flight motoneurons were labeled via the C380-GAL4, UAS-mCD8-eGFP; Cha-GAL80 driver [89] that drives the expression of GAL4-dependent, membrane-bound eGFP mostly in motoneurons for anatomical analysis. The Cha-GAL80 transgene was used to block GAL4 in cholinergic neurons, preventing GAL4-dependent manipulations to affect presynaptic cholinergic neurons. Dissection and analysis followed as we previously described in [98,99]. Briefly, flies were dissected in PBS, fixed in 4% paraformaldehyde, and mounted in glycerol [100]. Optical sections of motoneuron dendrites were acquired using identical settings with a 63× oil-immersion lens on a Leica SP5 confocal laser scanning microscope. For each Z-stack, maximum intensity projections were created, and motoneuron arborizations were selected within the dendritic region of interest using the free-hand selection tool of Fiji/ImageJ software (v1.54f, NIH, Bethesda, MD, USA). GFP fluorescence was measured as the mean gray value within that region, including subtraction of background fluorescence as described in [98,99]. The flight assay was performed as we previously described in [98,101] based on the method described in [102] and as modified by Nelson et al. [103]. For NMJ analyses, third instar larvae were dissected, and neuromuscular synapses were analyzed as previously described in [104,105,106]. Whereas phalloidin has been used in previous publications [20] to counterstain muscle tissue, we visualized neuronal tissue and synaptic boutons using Goat anti-HRP (1:250; Jackson ImmunoResearch #123-005-021) and Cy5-conjugated Donkey anti-goat (1:250; Jackson ImmunoResearch #705-175-147). As described in previous publications [18,88], although ectopic contacts were observed throughout the musculature, the scoring and quantification of the miswiring phenotype were restricted to muscles 7 and 6 in the abdominal segments A2–A7 as previously described [19,20]. Although some anatomical differences in the number of native boutons between A2 and A7 abdominal segments have been observed, no particular differences in the anatomical features or frequency of ectopic contacts have been observed across abdominal segments. Frequency of ectopic contacts is presented as mean ± SEM.

### 2.5. Social Assay

We performed a social attraction assay modified from [107]. Briefly, sociality chambers were created using 60 mm petri dishes filled with Sylgard. Using UV glue, ten flies (five males and five females) were glued to one side of the dish acting as attractor flies in the “attached” condition. An empty (no flies attached) Sylgard dish was used in the “empty” condition. Ten 4- to 5-day-old free walking flies of the same sex were transferred to the dishes. After 30 min of habituation, the movement of the flies in the dish was recorded every 15 s for 3 h. Social attraction was defined as the proportion of times the free-walking flies on the side of the immobilized flies. For each frame, the preference index (PI) was calculated as follows: PI = (total # of free walking flies − # of free walking flies on the side without attached flies)/total # of free walking flies. Thus, PI > 0 indicates attraction, whereas PI < 0 repulsion. The averaged PI value for each run was calculated.

### 2.6. Proteomics Sample Preparation

Flies aged 3–5 days old were flash frozen on liquid nitrogen and spontaneously vortexed to remove their heads. Heads were removed from bodies and wings using No. 25 sieves (Cole-Parmer (Vernon Hills, IL, USA)). For mass spectrometry, three separate samples for each genotype were prepared. Each sample contains 25 heads from males and 25 heads from females mixed for 50 heads total in each sample. Each individual sample was run independently, and LFQ was performed using the three samples from each genotype combined. Proteomic analysis was performed on these three samples from each noted genotype that were collected, extracted, and digested in parallel on the same day. Proteomic analysis was not repeated on another set of samples for cross validation. The frozen heads were weighed, and RIPA (Thermo Fisher Scientific (Waltham, MA, USA)), supplemented with protease inhibitor cocktail (Roche Diagnostics GmbH (Mannheim, Germany)), was added at 7.5 µL per milligram of tissue and hand homogenized with mini pestles. Homogenate was incubated at 4 °C for 30 min followed by 20-min centrifugation (at 4 °C) to clarify the lysate. Supernatant was collected and quantified using a Pierce BCA protein assay kit (Thermo). Supernatant was stored at −20 °C until usage.

Samples were prepped for MS analysis using S-Trap micro MS sample prep kit (Protifi (Fairport, NY, USA)). For each genotype, three samples were prepared using 100 µg of lysate following the standard S-Trap kit protocol and digested overnight using Trypsin Lys-C Mix (Promega (Madison, WI, USA)) with 10 µg per sample added at a concentration of 0.5 µg per µL of S-Trap micro MS sample prep kit digestion buffer (Protifi (Fairport, NY, USA)). Peptides were eluted in standard kit buffers and frozen at −20 °C until dried down and reconstituted for injection.

### 2.7. Proteomics Mass Spectrometry Acquisition and Data Analysis

Samples were analyzed on a Thermo Eclipse mass spectrometer (San José, CA, USA) with FAIMS interface, coupled to an Ultimate3000 HPLC. Then, 5 μL (1.5 μg total) injections were loaded onto a PepMap 100, 75 μm id × 2 cm C18 trap column (Thermo Fisher Scientific) at 3 μL/min for 10 min with 2% acetonitrile (*v*/*v*) and 0.05% formic acid (*v*/*v*). Chromatographic separation was performed using an Easy-Spray 75 μm id × 75 cm length, 2 μm, 100 Å C18 column (Thermo Fisher Scientific) at 60 °C. Samples were resolved using a 200 min gradient that began with an increase from 5% to 10% mobile phase B (80% acetonitrile + 0.1% formic acid in water) over the course of 10 min, followed by a linear increase to 90% mobile phase B at 161 min at a flow rate of 300 nL/min. The Thermo Eclipse was operated in positive polarity and data-dependent mode (topN, 3 s cycle time) with a dynamic exclusion of 60 s (with 5 ppm error). FAIMS CVs were set to −50 V, −65 V, and −85 V, with the 3 s cycle time split between each CV. MS1 scan resolution using the Orbitrap was set at 240,000, and the mass range was set to 350 to 2000 m/z. Normalized AGC target was set to 250%, with maximum injection time set to auto. Monoisotopic peak determination was used, specifying peptides, and an intensity threshold of 5 × 10^3^ was used for precursor selection. Data-dependent MS2 fragmentation was performed using higher-energy collisional dissociation (HCD) at a collision energy of 28% with quadrupole isolation at 1 m/z width. Ion trap scan rate was set to turbo, with a maximum injection time of 35 ms.

Untargeted proteomics data analysis was performed with Thermo Scientific Proteome Discoverer (v2.5) [108]. Database search was performed with Sequest [109] against the *Drosophila melanogaster* canonical and isoform fasta (UP000000803) downloaded from Uniprot [110] (15 December 2024) with an MS1 tolerance of 10 ppm and MS2 tolerance of 0.6 Da. A variable protein N-terminal acetylation was also included in the search along with oxidation on methionines and a static carbamidomethylation on cystines. FDR control was achieved utilizing the Percolator [111,112] node with 0.01 and 0.05 cutoffs for high and medium confidence. PTM localization was performed with IMP-ptmRS [113]. Label free quantitation was performed with the Minora feature detection node.

Visualization of PPI pathway enrichment was performed in Metascape [114] (v3.5) and edited using Cytoscape [115] (v3.10.3). Selected gene interaction visualization was performed in Cytoscape (v3.10.3) using selected genes input with a 0.4 confidence cutoff and a maximum of 8 additional interactors. Edge length was calculated using the STRING confidence score and was set equal to 1 − confidence score, where the longest edges will be the most distant interactions and the shortest will be the more confident direct interactions.

Mass spectrometry data were deposited in PRIDE under dataset identifier PXD061426.

### 2.8. Statistical Analysis

We used GraphPad Prism v10.4.1 (GraphPad Software Inc., San Diego, CA, USA) for all our statistical analyses unless noted otherwise. Descriptive statistics and normality distribution tests were carried out to assess the appropriate statistical analysis on all data collected. For sleep behavior, under light/dark (LD) conditions, the number of sleep bouts and total sleep time were compared using ANOVA, while Mann–Whitney U tests were used for comparing non-parametric data such as average sleep bout length. Circadian rhythm analyses under LD and constant darkness (DD) conditions included comparisons of period length and rhythmicity strength (power–significance threshold) using Mann–Whitney tests, and Fisher’s exact test was applied to rhythmicity index (RI) frequency distributions. In the social behavior assay, the preference index (PI) was analyzed using a *t*-test to compare control and *CaMKI*^LOF^ flies under “attached” versus “empty” conditions. For aging-dependent flight performance, comparisons across age groups (2, 10, and 30 days) were made using the Kruskal–Wallis test, a non-parametric alternative to ANOVA, due to non-normal data distribution. Dendritic fluorescence intensities were analyzed using one-way ANOVA to detect differences between control and *CaMKI*^LOF^ flies. At the larval neuromuscular junction (NMJ), frequencies of ectopic synaptic contacts were analyzed using one-way ANOVA or the Kruskal–Wallis test depending on data distribution. For proteomic and phosphoproteomic analyses, volcano plots were generated using adjusted *p*-values (FDR ≤ 0.05) and log2 fold changes, with statistical significance assessed via Proteome Discoverer software tools. To summarize, the parametric *t*-test, ANOVA, and Mann–Whitney multiple *t*-test, or the non-parametric Kruskal–Wallis test, were used to compare experimental genotypes to their respective controls as described in the corresponding figure legends.

No artificial intelligence software was used at any time for the writing of the manuscript or data analysis.

## 3. Results

### 3.1. Loss of *CaMKI* Increases Sleep Duration and Alters Sleep Architecture in Drosophila

**Figure 1:**
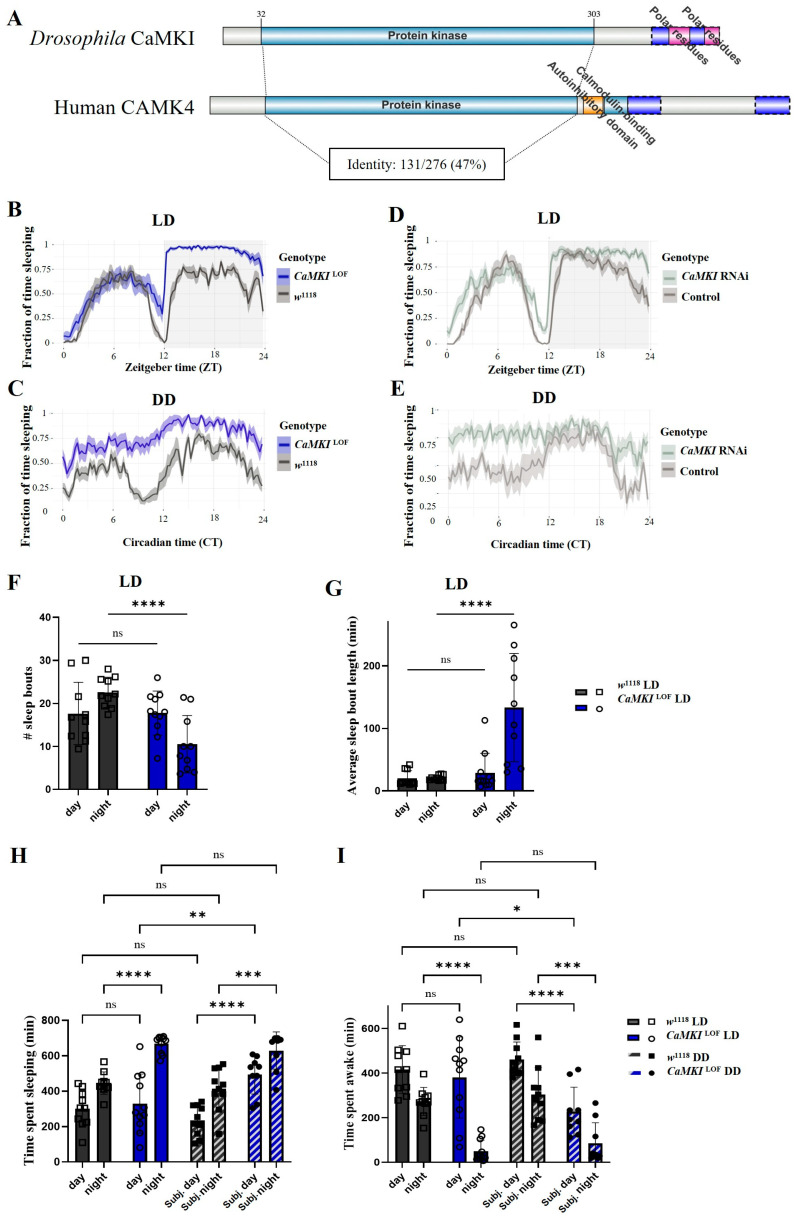
*CaMKI* dysregulation affects sleep behavior. (**A**) IBS2.0 protein domain map of *Drosophila* CaMKI (Q7JMV3) and human CAMK4 (Q16566). Domains annotated based on UniProt entries, on human CAMK4, there is a region of multiple domain overlap between an Autoinhibitory domain (residues 305–321), a PP2A-binding domain (not depicted on the diagram) (residues 306–323), and a Calmodulin-binding domain (residues 322–341). Identity of domains calculated from alignment of CaMKI (NP_524622.1) and CAMK4 (NP_001310303.1) on DIPOT (v9). (**B**) Sleep fraction over a 24-h period in 12 h:12 h light–dark (LD) conditions for *CaMKI* mutants (*CaMKI*^LOF^, blue; *n* = 11) and control (*w*^1118^, black; *n* = 10) flies. Shaded regions represent the standard error of the mean (SEM). (**C**) Sleep fraction over a 24-h period in 12 h:12 h dark–dark (DD) conditions for *CaMKI*^LOF^ and *w^1118^* males. (**D**) Sleep fraction over a 24-h period in LD conditions for *CaMKI*_RNAi (light-gray; elav^c155^-Gal4 > *CaMKI*_RNAi; BDSC_35362; *n* = 11) and genetic controls (gray; elav^c155^-Gal4 > *w^1118^*; *n* = 10) males. (**E**) Sleep fraction over a 24-h period in DD conditions for *CaMKI* RNAi and control flies. (**F**,**G**) Quantification of number of sleep bouts and average sleep bout length in each genotype under LD conditions. (**H**,**I**) Total time spent sleeping and time spent awake comparing genotypes, control and *CaMKI*^LOF^, under LD and DD conditions. Error bars represent ±SEM. Statistical significance is indicated (* *p* < 0.05, ** *p* < 0.01, *** *p* < 0.001, and **** *p* < 0.0001). Statistical analysis was performed using ANOVA to compare number of sleep bouts and time spent sleeping, while non-parametric multiple comparison Mann–Whitney *t*-test was performed to compare average sleep bout length between genotypes. The analysis of sleep parameters for DD condition was carried out averaging the data in a 24 h cycle and not based on the observed genotype period cycle.

The *CAMK4* (calcium/calmodulin-dependent protein kinase 4) is considered a risk gene for ASD [36,37,38,39,40], but the molecular mechanisms linking *CAMK4* dysregulation and ASD-associated phenotypes remain poorly understood. Therefore, we used *Drosophila melanogaster* as a model system to investigate ASD-related phenotypes in flies with dysregulated *CaMKI*, the fly homolog of mammalian *CAMK4*. *Drosophila* CaMKI displays 47% identity and 62% similarity to the human CAMK4 protein (based on the DIOPT ortholog mapping online resource [116]). The CaMK family of proteins was initially defined by the presence of an intrinsic regulatory Ca^2+^/calmodulin-dependent (CaM) domain; however, it is now well documented that many family members either do not contain this domain or are not Ca^2+^/calmodulin-dependent [117]. Evolutionarily, human CAMK1 and CAMK4 are closely related as defined primarily by sequence of catalytic domain supplemented with information regarding biological function [118,119]. Consistently, compared to human CAMK4, *Drosophila* CaMKI shows a highly conserved sequence of the catalytic domain, lacking the CaM binding domain (Figure 1A). The biological role of *CaMKI* in *Drosophila* is not well studied, likely due to the challenges associated with its location on the fourth chromosome [120,121,122], but its transcriptional role in Or47b neurons to modulate pheromone detection and courtship behavior in males is well established [41,42].

Sleep is a highly conserved biological process regulated by both circadian rhythms and homeostatic mechanisms [47,123]. Sleep disturbances are common to neurodevelopmental and neurodegenerative conditions and are thought to contribute to cognitive impairment, behavioral issues, and disease progression [124,125]. It is extensively documented that ASD patients experience altered sleep behavior, with the majority reporting insomnia, fragmented sleep patterns, and longer sleep onset, all generally leading to less sleep duration, while others experience hyper-insomnia, difficulties waking up, and longer sleep duration [126]. As many ASD patients suffer from sleep disturbances, we aimed to assess the role, if any, of *CaMKI* in sleep behavior by comparing the sleep patterns of *CaMKI*^LOF^ flies to controls (Figure 1B,C). In *Drosophila*, sleep is defined as five or more minutes of inactivity in which flies show an increased arousal threshold [95]. Sleep parameters were assessed under 12-h light–12-h dark (LD) cycles and constant darkness (DD) to examine sleep homeostasis. *CaMKI*^LOF^ mutants exhibit an increased fraction of time spent sleeping during the dark phase in LD conditions (Figure 1B). *CaMKI*^LOF^ mutants appeared to struggle with wake time anticipation as the fraction of time sleeping in the control groups starts decreasing earlier than the mutant groups, suggesting that the mutant groups might rely more on the light cue to wake up rather than on their internal clock (Figure 1B).

Additionally, these mutants failed to maintain the typical bimodal circadian rhythm seen in controls under DD conditions (Figure 1C). Sleep fraction analysis revealed that *CaMKI*^LOF^ flies exhibited significantly decreased number of sleep bouts during the LD dark phase, but increased sleep bout length and time spent sleeping at night compared to controls (Figure 1F–H). Under constant darkness (DD) conditions, *CaMKI*^LOF^ flies exhibited increased sleep during the subjective day relative to light–dark (LD), indicating a disruption in circadian regulation and a diminished capacity to sustain endogenous rhythmicity compared to controls (Figure 1H,I). Sleep behavior and circadian rhythm were also assessed in flies expressing pan-neuronal RNAi-based *CaMKI* knockdown and compared to their genetic control (see Figure 1D,E; Supplementary Figure S1). Consistently, *CaMKI*-RNAi flies showed similar trends to the sleep defects observed in the corresponding mutant genotype (Figure 1D,E; Supplementary Figure S1).

Since sleep is estimated based on inactivity periods, we measured the activity index, which indicates the level of activity of the fly, in order to test whether the observed sleep phenotype is a consequence of broader motor dysfunction or actual sleep-specific effects. We observed no difference in the level of activity between *CaMKI*^LOF^ mutants, *CaMKI*-RNAi flies, and their control groups, suggesting that the phenotype we observed was likely due to sleep/circadian disruptions and not motor dysfunction, overall fitness, or health conditions (Supplementary Figure S2). Overall, the data show that flies with dysregulated *CaMKI* exhibited altered sleep behavior, with fewer sleep bouts but longer sleep durations and increased time spent sleeping at night compared to control flies under normal light/dark conditions. The decreased number of sleep bouts and longer sleep duration during nighttime compared to control in our study suggests higher-arousal threshold and, therefore, altered homeostatic regulation, which is consistent with some of the sleep disturbances observed in ASD individuals [126].

Furthermore, *CaMKI*^LOF^ mutants displayed increased subjective daytime sleep under constant darkness. The nearly complete loss of the difference in sleep time between subjective day and subjective night that we observed in *CaMKI*^LOF^ under constant dark conditions (Figure 1D), and, therefore, the difficulties of the mutant flies to maintain a bimodal pattern of activity independent of the presence of light cues, offered partial insights on the rhythmicity strength of these flies. Therefore, we proceeded to investigate circadian rhythmicity further.

### 3.2. *CaMKI* Dysregulation Disrupts Circadian Locomotor Rhythms

**Figure 2:**
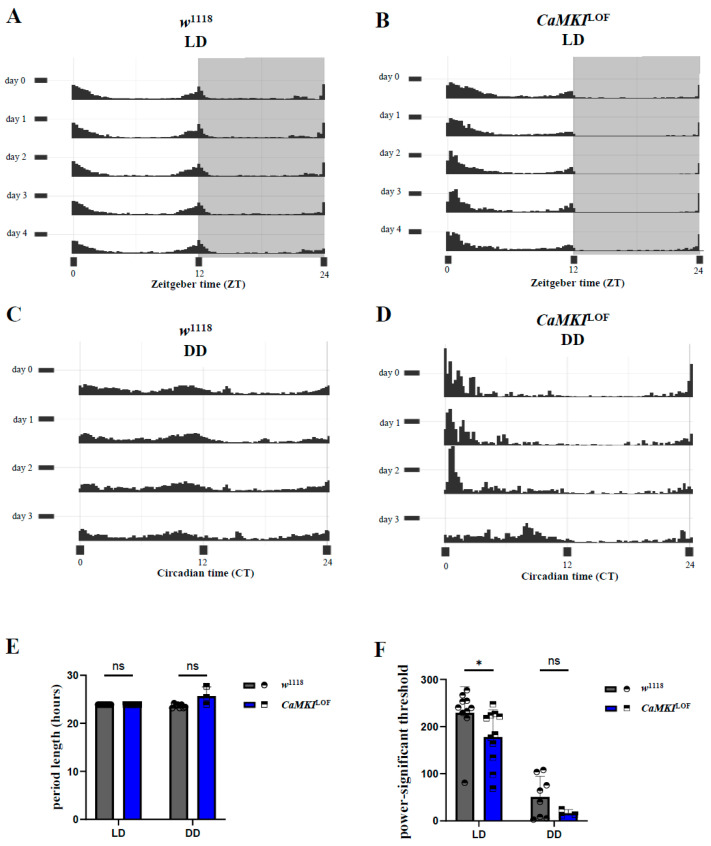
Circadian rhythm is disrupted following *CaMKI* dysregulation. (**A**) Actograms of *w*^1118^ control males over a 48-h period in LD conditions. White and shaded gray areas represent light and dark phases, respectively. (**B**) Actograms of *CaMKI*^LOF^ males in LD conditions over 48 h, showing altered activity patterns compared to controls. (**C**) Actograms of *w*^1118^ males in constant darkness (DD) over 24 h, displaying sustained rhythmic activity. (**D**) Actograms of *CaMKI*^LOF^ males in DD conditions, showing disrupted rhythmic activity. (**E**,**F**) Quantification of circadian period length and power–significance threshold under LD and DD conditions. Bars represent mean + SEM. Statistical significance is indicated (* *p* < 0.05). Statistical analysis was performed using the non-parametric Mann–Whitney test for multiple *t*-tests to compare circadian parameters between genotypes.

To assess whether *CaMKI* dysregulation affected circadian rhythm, we analyzed the circadian strength under LD (Figure 2A,B) and DD (Figure 2C,D) conditions as described in previous studies [95]. Additional analysis on circadian rhythmicity revealed a disrupted internal clock regulation in *CaMKI*^LOF^ mutants. First, the number of rhythmic flies, determined through chi-square analysis of periodograms (see methods), revealed how *CaMKI*^LOF^ mutants were less rhythmic compared to the control flies in the absence of light cues (% rhythmic flies—LD: control flies → 100%, *CaMKI*^LOF^ → 100%; DD: control flies → 80%, *CaMKI*^LOF^ → 33%). Then, control flies exhibited robust morning and evening anticipatory activity peaks under LD, while *CaMKI* mutants displayed attenuated morning anticipation (Figure 2A,B). Although *CaMKI*^LOF^ mutants exhibited the same circadian period length as control flies, their circadian strength—measured by the power–significance threshold (see methods) (Figure 2E,F) and the rhythmicity index—was significantly lower under standard 12h:12h light/dark conditions, indicating a weaker internal regulatory mechanism in the mutants. Consistently, *CaMKI*-RNAi flies showed a similar effect on circadian regulation as the mutant genotype (Supplementary Figure S1). Together, these findings demonstrate that *CaMKI* dysregulation disrupts normal sleep homeostasis, intended as the regulation and balancing of sleep-to-wake states, and circadian rhythm regulation in *Drosophila*. The observed increase in sleep duration and impaired circadian rhythmicity suggests that *CaMKI* plays a critical role in neuronal circuits governing sleep–wake transitions and endogenous rhythmicity.

### 3.3. Social Behavior Is Affected in Adult *CaMKI*^LOF^ Mutant Flies

**Figure 3:**
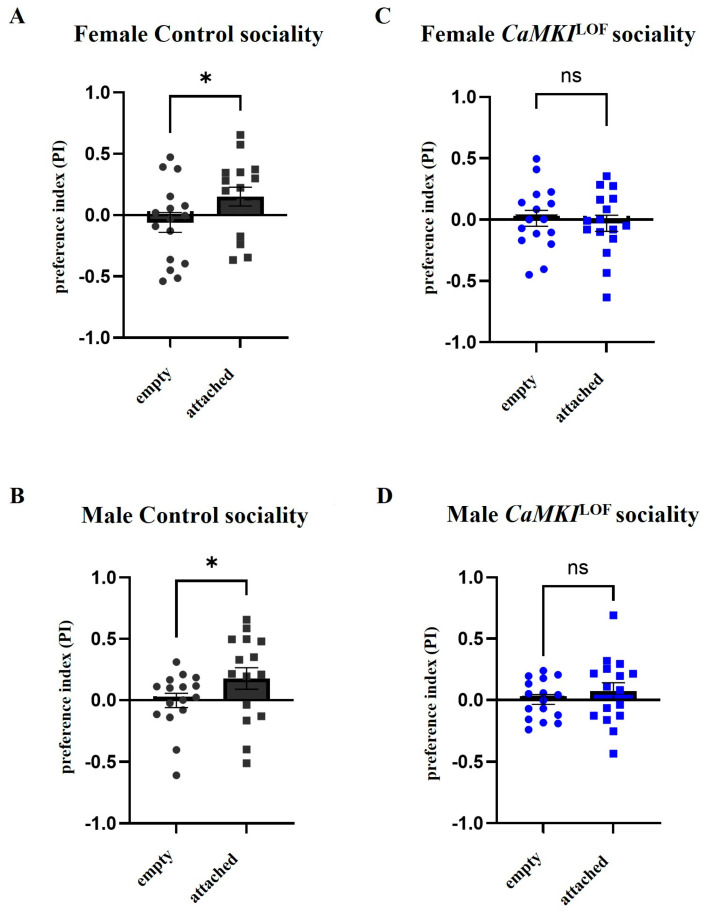
Social attraction is disrupted in *CaMKI*^LOF^ mutants. Quantification of social approach behavior by calculating the preference index of free-walking flies as they are attracted to immobilized flies on a dish (attached condition; squares) as compared to their response on a dish with no immobilized flies (empty condition; circles) in *w*^1118^ controls ((**A**,**B**); dark gray) and *CaMKI*^LOF^ mutants ((**C**,**D**); blue). Whereas social attraction was observed in control females (**A**) and males (**B**), no social attraction was observed in female (**C**) or male (**D**) *CaMKI*^LOF^ mutants. Each dot indicates the mean preference index (PI) from a run containing 10 free-walking flies (*n* = 16 runs for each group). Bars indicate mean ± SEM. Statistical analysis was performed using *t*-test (* *p* < 0.05).

As deficits in social interaction is one of the main criteria for diagnosing ASD, we used a recently developed paradigm to study social approach behavior [107] to examine sociality interactions in *CaMKI*^LOF^ mutants. For this, we measured social attraction when 10 flies were tethered to one half of a shallow circular chamber to serve as attractor flies and measure whether free-walking flies spend significantly more time on the same side as attractors. By contrast, free-walking flies exhibit no bias to either side of the chamber in an empty chamber with no attractor flies. As shown in Figure 3, control females (Figure 3A) and males (Figure 3B) spent significantly more time on the same side as attractors, whereas no bias to either side was observed in an empty chamber, indicating that control flies had a strong tendency to associate with other flies, as previously described [107]. Interestingly, *CaMKI*^LOF^ males or females (Figure 3C,D) showed no bias to either side of the chamber in the presence of the attached flies or in an empty chamber, suggesting no tendency for sociality. No additional anxiety-like, repetitive behaviors (e.g., grooming) were observed, although this was not rigorously quantified.

### 3.4. *CaMKI* Dysregulation Enhances Behavioral Decline and Neurodegeneration

**Figure 4:**
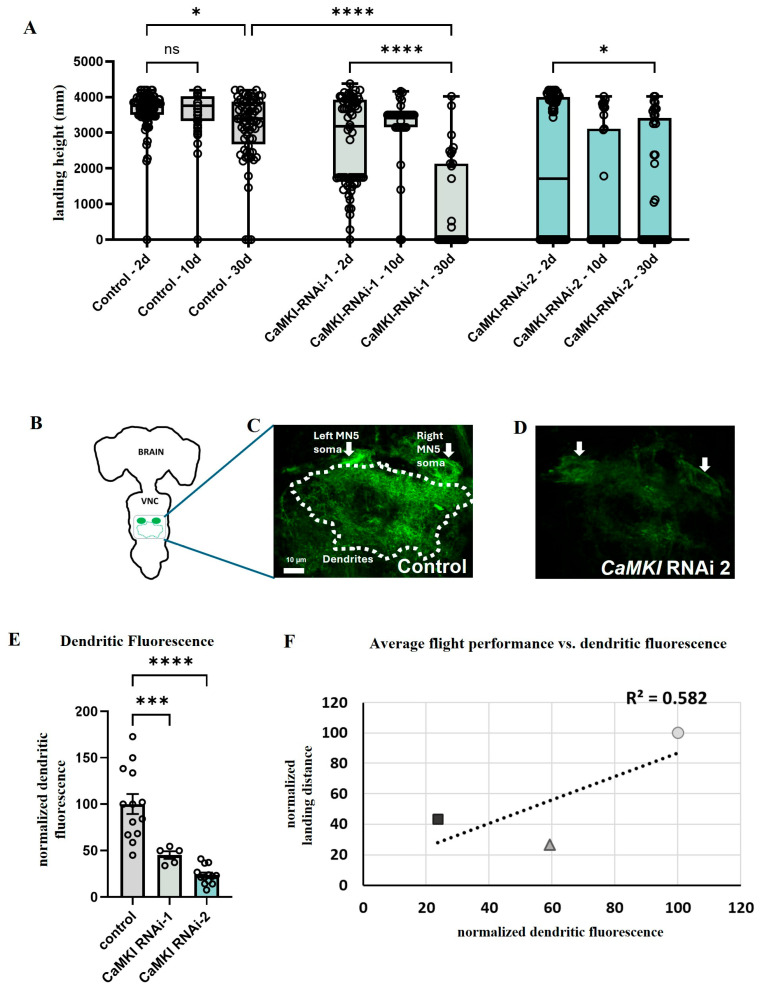
*CaMKI* dysregulation enhances behavioral decline and dendritic degeneration in aging flies. (**A**) Expression of *CaMKI*-RNAi in adult motoneurons controlling wing muscles (using the C380-GAL4 driver) resulted in severely impaired flight response, as assessed by the landing distance in 2, 10, and 30-day-old males from controls (C380-GAL4, UAS-CD8-GFP/Y; Cha-GAL80/+; *n* = 99, 43, 63 flies for 2, 10, and 30 day-old) and in *CaMKI* knockdown flies (CaMKI-RNAi-1: BDSC_41900, *n* = 64, 34, 35 flies for 2, 10, and 30 day-old; CaMKI-RNAi-2: BDSC_35362, *n* = 62, 39, 41 flies for 2, 10, and 30 day-old). Data are presented as median and quartiles. Statistical analysis was performed using Kruskal–Wallis test. (* *p* < 0.05, **** *p* < 0.0001) (**B**) Schematic showing an adult brain and ventral nerve cord (VNC) in addition to the location of motoneurons that innervate the flight muscle. Green circles indicate cell bodies of the identified motoneuron MN5 with the area of the dendritic fields represented by the green dotted line. Dotted square indicates area of pictures shown in (**C**,**D**). (**C**,**D**) Maximum projection images from confocal stacks showing the two MN5 somata (arrows) and the dendritic area from all 10 DLM motoneurons (dotted line) in control (C380-GAL4, UAS-CD8-GFP/Y; Cha-GAL80/+) and in *CaMKI* knockdown males (C380-GAL4, UAS-CD8-GFP/Y; Cha-GAL80/UAS-CaMKI-RNAi2-BDSC_35362) both from 30-day-old flies. (**E**) Quantification of dendritic fluorescence normalized to the baseline fluorescent value in control flies vs. the two different groups of *CaMKI* knockdown flies as shown in (**C**,**D**) (*n* = 13, 3, 13). Error bars represent ± SEM. Statistical analysis was performed using one-way ANOVA. (*** *p* < 0.001, **** *p* < 0.0001) (**F**) Correlation of landing height (normalized to control) with dendritic fluorescence (normalized to control mean gray values) from 30-day-old flies of controls (circle), CaMKI-RNAi-1 (triangle) and CaMKI-RNAi-2 (square), indicating that poor flight performance correlated with decreased dendritic fluorescence as we previously described [99].

A link between autism and aging-dependent conditions such as dementia and Alzheimer’s disease has been proposed in numerous recent studies [53,54,55,56,57]. We took advantage of the strengths of the *Drosophila* model for aging research [58] to investigate the effects of *CaMKI* dysregulation on aging-dependent processes. We focused on the motor network and examined locomotion performance, which is also consistent with the prevalence of motor dysfunction in ASD patients [60,61] and in other ASD animal models [8]. For this, we quantified the decline in flight performance as behavioral readout as well as the intensity of dendritic fluorescence in adult flight motoneurons as a means of anatomical integrity as previously described in our publications [83,98,99,101,127,128] and as used in anatomical studies of mammalian brains [129]. Interestingly, the decline in flight performance was significantly more pronounced in flies expressing *CaMKI* RNAi-knockdown in adult motoneurons than their corresponding controls (Figure 4A). This locomotion defect was phenocopied using a second RNAi construct. We next quantified the relative fluorescence intensity of the dendritic field in controls and in *CaMKI*-knockdown flies to test for changes in dendritic anatomy (Figure 4B–E). Consistent with our behavioral data, flies expressing either of the two RNAi constructs to knock down *CaMKI* showed a significant decrease in dendritic fluorescence as compared to controls (Figure 4E), suggesting that *CaMKI* dysregulation may enhance aging-dependent dendritic degeneration. Thus, a strong correlation between poor flight performance and increased dendritic degeneration was observed (Figure 4F). In contrast to *CaMKI* knockdown, such a decrease in dendritic fluorescence and flight performance was not observed in flies expressing different RNAi constructs that target other genes not involved in degeneration that we analyzed for a different study.

### 3.5. Loss of *CaMKI* Affects Synaptic Development Increasing Ectopic Synapses

**Figure 5:**
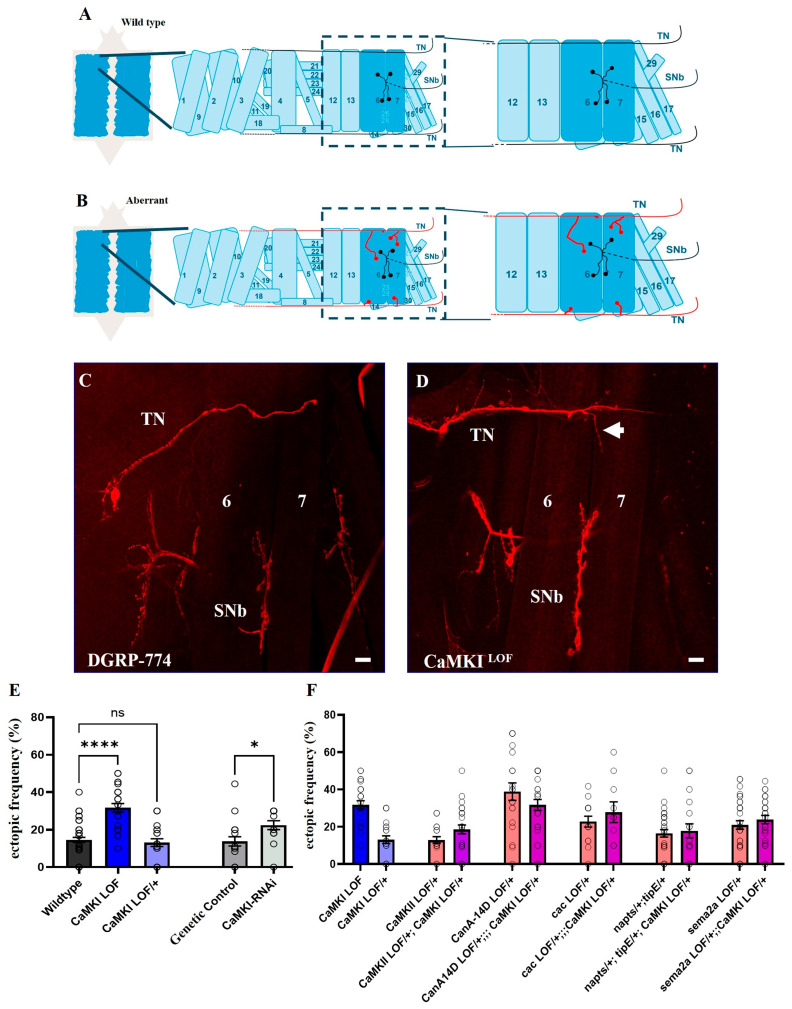
*CaMKI* disruption leads to NMJ miswiring. (**A**,**B**) Schematic diagrams of the transverse nerve (TN) and segmental nerve b (SNb) motor neuron innervations, illustrating their structural organization, key branch points, and synaptic contacts on target muscles. The upper schematic represents the control condition in which the native SNb motor neurons innervate muscles 6 and 7, while the TN passes muscles 6 and 7 without innervating them. The lower schematic highlights alterations (red markings) observed under specific genetic perturbations, where the TN presents innervations and ectopic synaptic contacts onto muscles 6 and/or 7. (**C**,**D**) Confocal images of TN and SNb axon projections and synaptic boutons on muscles in controls (DGRP-774) and *CaMKI*^LOF^ mutants. In *CaMKI*^LOF^ mutants (**D**), the TN shows ectopic synaptic contacts onto muscle 7 (white arrow). Scale bars: 20 µm. (**E**) Frequency of ectopic contacts in controls (*n* = 51), *CaMKI*^LOF^ homozygotes (*n* = 25), and heterozygotes (*n* = 22), as well as in larvae expressing pan-neuronal *CaMKI*-RNAi BDSC_35362 (*n* = 14) and its corresponding genetic control (elav^C155^-Gal4/*w^1118^*; *n* = 19). Bars represent mean values ± SEM with statistical significance indicated. (**F**) Quantification of ectopic innervations under different genetic conditions for genetic interaction tests involving genes previously identified to regulate activity-dependent synaptic refinement [18,19,20] (*n* = 25, 22, 20, 26, 20, 25, 54, 10, 34, 18, 34, and 29). Bars represent mean values ± SEM with statistical significance indicated. Statistical analysis was carried out performing one-way ANOVA and non-parametric Kruskal–Wallis test depending on data distribution (* *p* < 0.05, **** *p* < 0.0001).

Aberrant synaptic refinement during early development has been proposed to underlie the synaptic phenotypes observed in brains of autistic patients [1,2,3]. To investigate whether *CaMKI* disruption affects synaptic anatomy, we used the well-characterized stereotypic anatomy of larval neuromuscular junction (NMJ) to examine changes in synaptic connectivity. During early embryonic development, motoneuron growth cones extend their filopodia and innervate not only their target muscles but also off-target muscle fibers [130,131,132]. These temporary off-target contacts have to be removed in an activity-dependent manner, otherwise they are stabilized to become functional ectopic synapses [13,18,19,88] (Figure 5A,B). Compared to control larvae (Figure 5C), *CaMKI* mutants showed an elevated frequency of ectopic contacts (Figure 5D,E). Pan-neuronal expression of an RNAi construct to knock down *CaMKI* phenocopied miswiring, leading to increased ectopic frequency in knockdown animals as compared to controls (Figure 5E). As part of the activity-dependent mechanism regulating synaptic refinement, recent studies have identified numerous molecules acting downstream of neuronal activity and calcium (Ca^2+^). These include the Ca^2+^-dependent adenylyl cyclase Rutabaga [20], the Ca^2+^/calmodulin-dependent serine/threonine kinase CaMKII [18,19], and its counterpart, the protein phosphatase Calcineurin (CaN) [19]. These activity-dependent molecules are thought to act downstream of presynaptic Ca^2+^ and cAMP oscillations to regulate the neuron’s responsiveness to chemorepulsion exerted by the postsynaptic muscle via the secreted chemorepellent Sema2a and its PlexB receptor [13,18,19,20]. Therefore, we performed genetic interaction tests to examine whether the molecular components identified in this pathway may functionally interact with *CaMKI*. For this, we followed a well-established method of using various heterozygous mutants used in previous publications that provide a sensitized genetic background to test for interactions with *CaMKI*^LOF^ heterozygotes, including *CaMKII*^LOF^/+, *CanA14D*/+, *cac*^LOF^/+, *nap^ts^*/+ *TipE*/+, and *Sema2a*/+ heterozygotes [18,19,20,88]. These genetic interaction assays test functional relationships between the molecules, pointing to an epistatic interaction when non-additive effects of their individual effects are observed. As shown in Figure 5F, control-like levels and no significant enhancement of the ectopic contact phenotype were observed in any of the double heterozygotes (Figure 5F), suggesting that *CaMKI* may be acting in a different molecular pathway to regulate synaptic connectivity (see below).

### 3.6. Proteomic Analyses Reveal Proteins Dysregulated by CaMKI Loss

**Figure 6:**
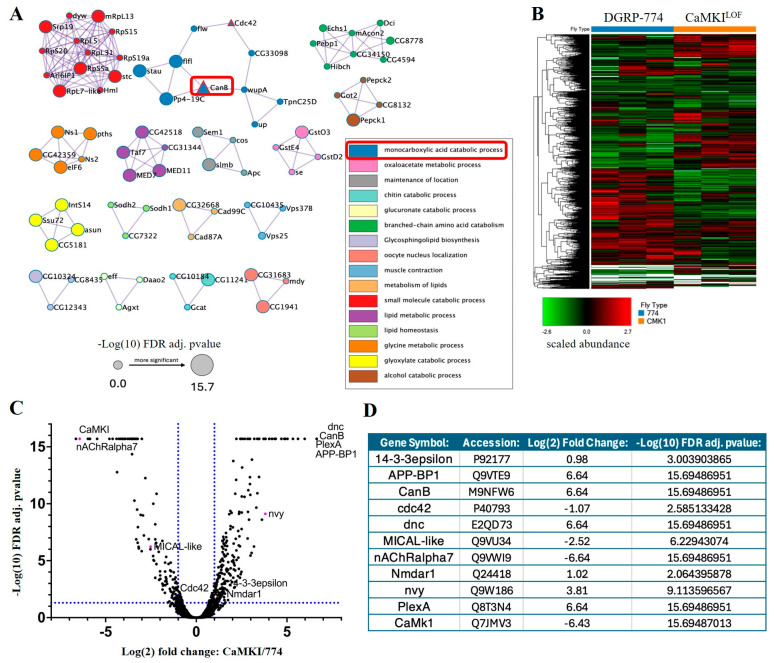
Proteome dysregulation is observed in *CaMKI* mutants. Data represent proteome-wide comparisons between *CaMKI*^LOF^ mutants and DGRP-774. (**A**) Metascape v3.5 [114] visualization of significant differentially expressed proteins (*p* < 0.05) using protein–protein interaction (PPI)-grouped pathways. Genes of interest noted by red bordered triangles. Various pathways associated with clustering listed in legend and defined in *S3_SUPP—Metascape GO all PPl* (**B**) Clustering of proteome in samples of each genotype visualizing scaled protein abundance. Created using Proteome Discoverer. (**C**) Volcano plot of differentially expressed proteins between *CaMKI*^LOF^ mutants and DGRP-774 flies. Proteins of interest highlighted in magenta with name adjacent. Generated in GraphPad Prism 10 using abundance ratios and adjusted significance values generated in Proteome Discoverer. Full protein target list and associated values available in *S4_SUPP—allproteomics data*. (**D**) Table listing proteins of interest along with fold change and significance values highlighted in panel (**C**).

**Figure 7:**
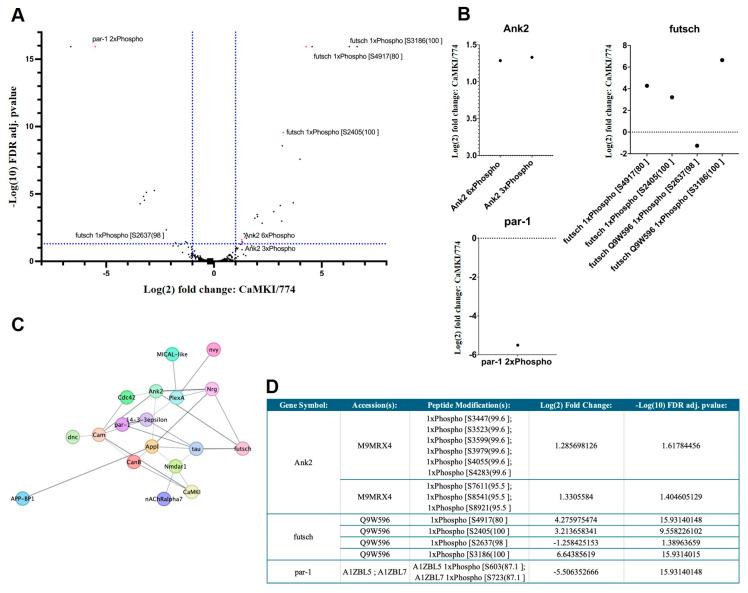
Phosphorylation differences are observed in *CaMKI*^LOF^ mutants. (**A**) Volcano plot of differentially expressed phospho-peptides in *CaMKI*^LOF^ mutants compared to DGRP-774. Peptides of interest highlighted in magenta with gene name and modification listed. Plot created in GraphPad Prism 10 using data generated from Proteome Discoverer, all phospho-peptide data available in *S5—phospho all data*. (**B**) Relative abundances of selected peptides of interest in *CaMKI*^LOF^ and DGRP-774. Quantified using Proteome Discoverer and visualized using GraphPad Prism 10. Graphs show fold change. (**C**) Protein–protein interaction network of candidate genes identified from proteomic and phosphopeptidomic analyses (Cytoscape v3.10.3). Nodes represent proteins, and edge length is proportional to 1—STRING confidence score, where weaker interactions appear more distant, using a 0.4 confidence cutoff and maximum of 8 additional interactors. (**D**) Table of selected peptides and associated fold change with significance (all data available in S5_SUPP—phospho all).

Finally, to further elucidate molecular pathways interacting with *CaMKI* in the regulation of the processes described above, we performed shotgun proteomics of *CaMKI*^LOF^ mutants. Our analysis showed that 639 protein accession numbers were identified to display significant changes in protein levels (FDR adj. *p*-value ≤ 0.05; Figure 6; including the expected decrease in CaMKI levels confirming the *CaMKI*^LOF^ mutant background) as well as in phosphorylation levels of peptides (Figure 7). All eight peptides detected for CaMKI are unique. In the *CaMKI* mutants, peaks are detected for some of these peptides suggesting that portions of the protein may be expressed. However, LFQ finds that CaMKI is downregulated significantly (Log(2) FC of −6.43 compared to control). Although the presence of CaMKI peptides does suggest portions of the protein are potentially present, the results in this study are able to show that the significant change in CaMKI is enough to generate both anatomical and behavioral phenotypes.

As the top candidate molecules dysregulated in *CaMKI* mutants, we identified the cAMP-phosphodiesterase Dunce (Dnc) and the phosphatase CanB (Figure 6C,D) that we have previously described to regulate synaptic refinement [19,20]. This result suggests the possibility of dynamic phosphorylation levels of target proteins to be regulated by an interplay between CaMKI-dependent phosphorylation and CanB-dependent dephosphorylation. Interestingly, we observed a significant change in phosphorylation levels in various molecules known to regulate cytoskeleton dynamics (Figure 7A–D), such as Futsch, Ankyrin2, and Par1. The microtubule associated protein Futsch (peptide modification: S4917(80), log2FC = 4.28; adj. *p* value = 15.93), a MAP1B-like protein required for dendritic and axonal development, is implicated in the regulation of microtubule dynamics [76] and is known to be phosphorylated by the GSK3beta-homolog Shaggy [133]. The cytoskeletal adapter Ankyrin2 (Ank2) is involved in neuronal morphogenesis [134], whereas Par1 is a kinase involved in cell polarity and microtubule stability [135] as well as in tau phosphorylation and toxicity [63]. Additional neuronal genes identified in our dataset known to regulate neuronal anatomy and cytoskeleton are the small GTPase Cdc42 that regulates actin cytoskeleton and cell polarity [75] and the neuronal Plexin A (PlexA) receptor known to promote axonal repulsion [77,78]. Interestingly, various molecules previously described to interact with PlexA also showed significantly different expression levels in our proteomic analyses. These include the actin regulatory oxidation–reduction enzyme MICAL-like that directly bind and disassemble actin filaments (F-actin) [79], the A kinase anchoring protein (AKAP) nervy that regulates repulsive axon guidance [80], and the phospho-binding protein 14-3-3ε necessary for PlexA-dependent repulsion and axon guidance [81]. Regarding degenerative processes, we also identified the β-amyloid precursor protein binding protein 1 (APP-BP1) to interact with CaMKI, providing a molecular link between ASD-associated phenotypes and aging-dependent degeneration due to its known interactions with Cullin-3 (Cul3), a ubiquitin ligase strongly associated with ASD [136,137]. APP-BP1 is also known to interact with the *Drosophila* β-amyloid precursor protein (Appl), a transmembrane protein associated with degeneration and phenotypes associated with Alzheimer’s disease (AD) [62,138,139].

## 4. Discussion

### 4.1. Drosophila as a Model to Study Autism-Associated Phenotypes

Autism spectrum disorder (ASD) refers to a broad range of conditions including challenges with social skills, repetitive behaviors, speech, and nonverbal communication [140,141]. Studies using animal models have investigated basic aspects of such complex behaviors, providing insight into fundamental aspects underlying ASD etiology [142,143,144]. The *Drosophila* genome is 60% homologous to that of humans and includes homologs of about 75% of genes associated with human diseases [145,146]. In particular, considering the strong genetic link to ASD, *Drosophila* has emerged as a valuable invertebrate model to expand our understanding of ASD-associated phenotypes and conserved molecular mechanisms [32,33,34,35]. Our study provides experimental evidence of loss-of-function mutations in the fly homolog *CaMKI* in ASD-associated phenotypes, providing initial steps to expand our understanding of potential roles of *CAMK4* in ASD-relevant pathways, paving the way for future research to confirm a clear etiology for CAMK4-related ASD. It is worth noting that, whereas monoallelic mutations in *CAMK4* have been identified in ASD patients, the *CaMKI*^LOF^ mutants used in our study showed strong phenotypic defects in homozygotes, while *CaMKI*^LOF^ heterozygotes showed control-like phenotypes. This apparent discrepancy can be explained by the functional consequences of the different allelic mutations as well as genetic regulatory mechanisms such as penetrance, expressivity, and pleiotropy that may differ between mutations, species, and individuals. This includes differences in epigenetic regulation of the homologous locus as well as compensatory effects by other genes in the required signaling pathways in a cell- or non-cell autonomous manner that may vary among species. Multiple examples of haploinsufficient mutations in genes leading to disease in humans without affecting heterozygous animals are well documented in validated mammalian models such as, for example, mutations in the zinc-finger transcription factors *GATA4* and *GATA6* associated with congenital heart defects in humans [147], mutations in the macrophage colony-stimulating factor receptor (*CSF1R*) associated with adult onset leukoencephalopathy [148], or mutations in the *PITX1* bicoid homeodomain transcription factor associated with clubfoot in humans [149]. Therefore, the insights provided in this study regarding *CaMKI* function in the nervous system may contribute to a better understanding of the fundamental functions of *CAMK4* and its association with ASD.

At the behavioral level, flies engage in various social behaviors including courtship, aggression, mating, sociality, and egg-laying, while the effects of group-housing vs. isolation are well documented [41,42,150]. Consistently, we observed disrupted sociality responses in *CaMKI*^LOF^ mutant females and males when compared to controls. Future follow-up studies will address the modulation of the observed *CaMKI*^LOF^ mutant phenotypes by different genetic backgrounds as well as by rescue experiments via functional *CaMKI* expression in *CaMKI*^LOF^ mutant flies. Furthermore, studies in *Drosophila* have examined the regulation of sleep [97,123,151], motor control [152,153,154], and neuronal activity [19,100,155], providing mechanistic insight into ASD-associated comorbidities such as sleep dysfunction, motor impairments, and epilepsy [60]. Consistently, *Drosophila* has been useful for characterizing the in vivo roles of ASD-associated genes and their regulatory networks involved in these behaviors [32,33,34,101,128,136,156]. We observed aberrant sleep and circadian patterns in *CaMKI*^LOF^ mutant flies, consistent with observations in ASD patients [48,52], which involve an increased number of awakenings during the night, sleep onset latency, and reduced sleep efficiency [157]. The sleep and circadian alterations that we observed suggest that *CaMKI* may play a role in the function of morning cells, a key circadian subgroup comprising the PDF-positive s-LNvs. The altered sleep patterns that we observed, including disrupted morning anticipation and increased sleep during the subjective day in constant darkness, mirror phenotypes previously reported in studies manipulating morning cells [158,159]. These parallels raise the possibility that *CaMKI* activity contributes to morning cell output or identity. Future studies should directly assess *CaMKI* function within identified circadian neurons to determine whether its loss specifically impairs morning cell signaling or broader circadian circuit dynamics.

At the anatomical level, postmortem studies of autistic patients that have mutations in genes associated with ASD showed increased synaptic density and longer dendritic spines [1,2,4,7], suggesting a link between ASD and aberrant synaptic refinement during early development. *Drosophila* has served as a suitable model to elucidate the molecular mechanisms underlying many aspects of anatomical remodeling including synaptic pruning [13] and dendrite regulation [160]. Given the highly conserved nature of basic signaling pathways, insight gained in *Drosophila* studies has the potential to be translated into novel ASD treatments by advancing our understanding of the mammalian and human brain.

### 4.2. Link Between Developmental Deficits and Aging-Dependent Degeneration

Although an ASD diagnosis in children is considered reliable at 24 months of age, several anatomical and behavioral differences can be observed between infants at high-risk for ASD and low-risk controls from age 6 to 12 months (reviewed in [161]). For example, using high-resolution brain magnetic resonance imaging (MRI), increased rates of surface area expansion from age 6 to 12 months, followed by an increased rate of total brain volume from 12 to 24 months, were detected [162]. Also, functional MRI (fMRI) studies showed reduced functional connectivity between the amygdala and canonical targets [163] and atypical connectivity in language areas [164] in children with ASD. Thus, consistent with the notion that ASD is considered a neurodevelopmental disorder, we observed behavioral changes in sleep, circadian rhythms, and sociality in young *CaMKI*^LOF^ mutant flies as well as miswiring indicating defects in synaptic refinement during the development of precise connectivity. On the other hand, recent studies indicated a higher prevalence of identified dementia diagnoses in ASD individuals [53,54,55,56,57]. Consistently, we observed experimental evidence of increased anatomical degeneration as well as enhanced behavioral decline in flies with disrupted *CaMKI*, suggesting that *CaMKI* may play an instructive role during development in addition to a neuroprotective role in aging flies or, alternatively, that *CaMKI*-dependent defects during development may promote aging-dependent degeneration. This is consistent with a strong cooccurrence of neurodevelopmental and neurodegenerative conditions that has been observed in other disease-related conditions such as, for example, in the well-characterized neurodevelopmental roles of the β-amyloid precursor protein (APP) associated with the formation of β-amyloid plaques and Alzheimer’s disease [139,165]. Interestingly, our proteomic results indicate an interaction between CaMKI and the β-amyloid precursor protein binding protein 1 (APP-BP1). Various molecular functions have been characterized for APP-BP1, including ubiquitin activation functions via interactions with Uba3 that in turn activates the ubiquitin-like protein NEDD8 in humans [166] and flies [62]. These interactions are required for neddylation of the ubiquitin ligase Cullin-3 (Cul3) [167], a gene strongly associated with ASD [136,137]. Moreover, during development, APP-BP1 is known to interact with the β-amyloid precursor protein-like (Appl) [62], the *Drosophila* ortholog for APP, a molecule associated with Alzheimer’s disease in humans [138]. Human APP and fly Appl share numerous conserved properties such as proteolytic processing, Aβ peptide formation, plaque buildup, memory regulation, and neurodegeneration (reviewed in [139,168,169,170]). However, the role of aberrant developmental processes in Alzheimer’s disease has not been considerably appreciated [139].

An additional link between neurodevelopment and degeneration is supported by our observation of significant changes in Par1 phosphorylation levels in *CaMKI* mutants. On the one hand, the Par1 kinase regulates synaptic development at the larval NMJ by phosphorylating scaffold proteins such as presynaptic Brp [171] and postsynaptic PSD-95/Dlg [172]. Regulation of anatomical changes in these processes can involve Par1 roles in regulating cell polarity and microtubule stability [135]. Additional structural roles include the Par1-dependent regulation of the cytoskeleton via interactions with the small GTPase Cdc42 [75] or with the microtubule binding protein Futsch, which has been proposed to be a likely substrate of Par1 [135] and shows decreased phosphorylation levels in our proteomic results. Future studies will examine the role of Par1 and Futsch in modulating the developmental defects observed in *CaMKI*^LOF^ mutants. Furthermore, Par1 is also involved in Tau phosphorylation and toxicity in *Drosophila* neurons [63], implicating its aberrant activation in the pathogenesis of AD and related tauopathies. Such roles of Par1 are highly conserved in its human homolog, the microtubule affinity regulating kinase (MARK), whose dysregulation has previously been associated with autism [173] as well as Alzheimer’s disease [174,175,176]. Therefore, our results provide initial mechanistic insight into the molecular means linking developmental processes and aging-dependent degeneration.

### 4.3. Molecular Pathways Involving *CaMKI* During Neurodevelopment and Degeneration

Our results indicate that dysregulation of *CaMKI* has various effects at the behavioral level, including aberrant sleep, circadian rhythms, sociality, and flight performance, in addition to cellular defects during synaptic development and neuronal network degeneration. Whereas most of the molecular interactions of CaMKI in the pathways regulating such processes remain to be tested, the results from our shotgun proteomic analysis revealed more than 600 proteins that have a statistically significant difference in the amount and phosphorylation levels in *CaMKI* mutants. This provides a list of various candidate genes and signaling pathways that may interact with CaMKI to regulate the biological processes described above.

In contrast to previous studies at the *Drosophila* NMJ that identified chemorepulsion exerted by the presynaptic receptor Plexin B as a key factor in synaptic refinement [18,19,20], the results from our genetic interaction tests with molecules involved in activity-dependent synaptic refinement indicated that CaMKI may regulate synaptic connectivity by acting in different molecular networks. From our proteomic data, we identified a group of genes that might represent an alternative chemorepulsion pathway regulating precise synaptic connectivity. These involve the neuronal Plexin A (PlexA) receptor, involved in axonal repulsion [77,78], the cytosolic flavoprotein MICAL, required for PlexA repulsive axon guidance [79], the phospho-binding protein 14-3-3ε, necessary for PlexA-mediated axon guidance and repulsion [81], and the A kinase anchoring protein (AKAP) Nervy (nvy), which antagonizes PlexA-mediated axonal repulsion [177]. Whether PlexA-mediated chemorepulsion may represent a separate pathway parallel to the PlexB-dependent pathway previously identified or, alternatively, whether there is a link between both pathways remains to be tested. The latter scenario is supported by our proteomic results showing dysregulation of the phosphatase CanB and the cAMP-phosphodiesterase Dunce (Dnc), both described in our previous studies to regulate PlexB-mediated synaptic refinement at the NMJ [19,20]. Whether a balance of phosphorylation levels between CaMKI-dependent kinase activity and CanB-dependent dephosphorylation regulates the phenotypes described above will be the focus of future research.

For the regulation of neuronal anatomy, an interesting set of genes worth highlighting include molecules known to regulate cytoskeleton dynamics, such as the microtubule associated protein Futsch [76], the cytoskeletal adapter Ankyrin2 [134] (Ank2; previously associated with ASD in a study using whole exome sequencing [40]), and the small GTPase Cdc42 [75] known to regulate actin cytoskeleton. This suggests molecular means for the CaMKI-dependent regulation of synaptic connectivity, which may involve the histone-acetyl transferase *CBP* and Juvenile Hormone [41,42], a hormone recently describe to regulate the maturation of naïve ethanol olfactory preference in young flies [178]. While most of the molecules described here are known to play a role during early stages of development, how *CaMKI* dysregulation affects aging-dependent degeneration remains an open question. As mentioned above, our proteomic analysis revealed changes in protein levels of APP-BP1 and phosphorylation levels of Par1 in *CaMKI*^LOF^ mutants. Thus, follow-up experiments will address the question whether the enhanced anatomical degeneration and behavioral decline are influenced by aberrant neurodevelopment or by molecular interactions between CaMKI, APP-BP1, Par1, and other molecules involved in aging and degenerative processes such as Appl.

## 5. Conclusions

Our data suggest that *CaMKI* plays a role in developmental processes and influences aging-dependent degenerative processes. These results will shed light on fundamental molecular pathways involving genes during developmental and degenerative processes, possibly providing new candidate genes for expanding our understanding of ASD etiology and the development of effective treatments.

## Data Availability

Mass spectrometry data deposited to PRIDE under dataset identifier PXD061426.

## Supplementary Materials

The following supporting information can be downloaded at https://www.mdpi.com/article/10.3390/biology14091228/s1: Supplementary Figure S1. CaMKI knockdown phenocopies effects on sleep and circadian rhythmicity. (A,B) Quantification of sleep parameters, including the number of sleep bouts and average sleep bout length (minutes), under 12 h:12 h light/dark (LD) conditions. (C,D) Time spent sleeping and time spent awake comparing genotypes, control, and knockdown under LD and DD conditions. Sleep was measured in genetic control (elav^c155^-Gal4 > *w*^1118^) and *CaMKI* RNAi (elav^c155^-Gal4 > *CaMKI*_RNAi; BDSC_35362) flies. Statistical analysis was performed using ANOVA to compare number of sleep bouts and time spent sleeping, while non-parametric multiple comparison Mann–Whitney *t*-test was performed to compare average sleep bout length between genotypes. The analysis of sleep parameters for DD condition was carried out averaging the data in a 24 h cycle and not based on the observed genotype period cycle. (E–H) Actograms showing sleep–wake activity rhythms over 24 h in LD and DD conditions. Genetic control and *CaMKI* RNAi flies are displayed separately. (I,J) Quantification of circadian rhythm parameters, including period length (hours) and rhythmicity strength defined as power–significance threshold, for genetic control and *CaMKI* RNAi flies in LD and DD conditions. White and shaded gray areas in LD graphs represent light and dark phases, respectively. Bars represent mean + SEM. Statistical significance is indicated (* *p* < 0.05). Statistical analysis was performed using the non-parametric Mann–Whitney test for multiple *t*-tests to compare circadian parameters between genotypes. Analysis performed for genetic control and *CaMKI* RNAi flies in LD and DD conditions. Supplementary Figure S2. Activity index suggests normal levels of activity in mutants compared to controls in LD conditions. Activity index (total awake time activity counts/total awake minutes) of different days (left y axis), plotted as violin plots, suggests no difference in levels of activity between *CaMKI*^LOF^ and *w*^1118^ (A), as well as between *CaMKI* RNAi and its control (B) under LD conditions. S3—SUPP_Metascape GOs all PPI Data excel. S4—SUPP_allProteomics_Data. S5—SUPP_phospho all.
