# Supplementary figures and images for: Dysregulation of Protein Kinase CaMKI Leads to Autism-Related Phenotypes in Synaptic Connectivity, Sleep, Sociality, and Aging-Dependent Degeneration in Drosophila
Source: Biology (Basel). 2025 Sep 9;14(9):1228. doi: 10.3390/biology14091228 (PMC12467204; doi:10.3390/biology14091228)

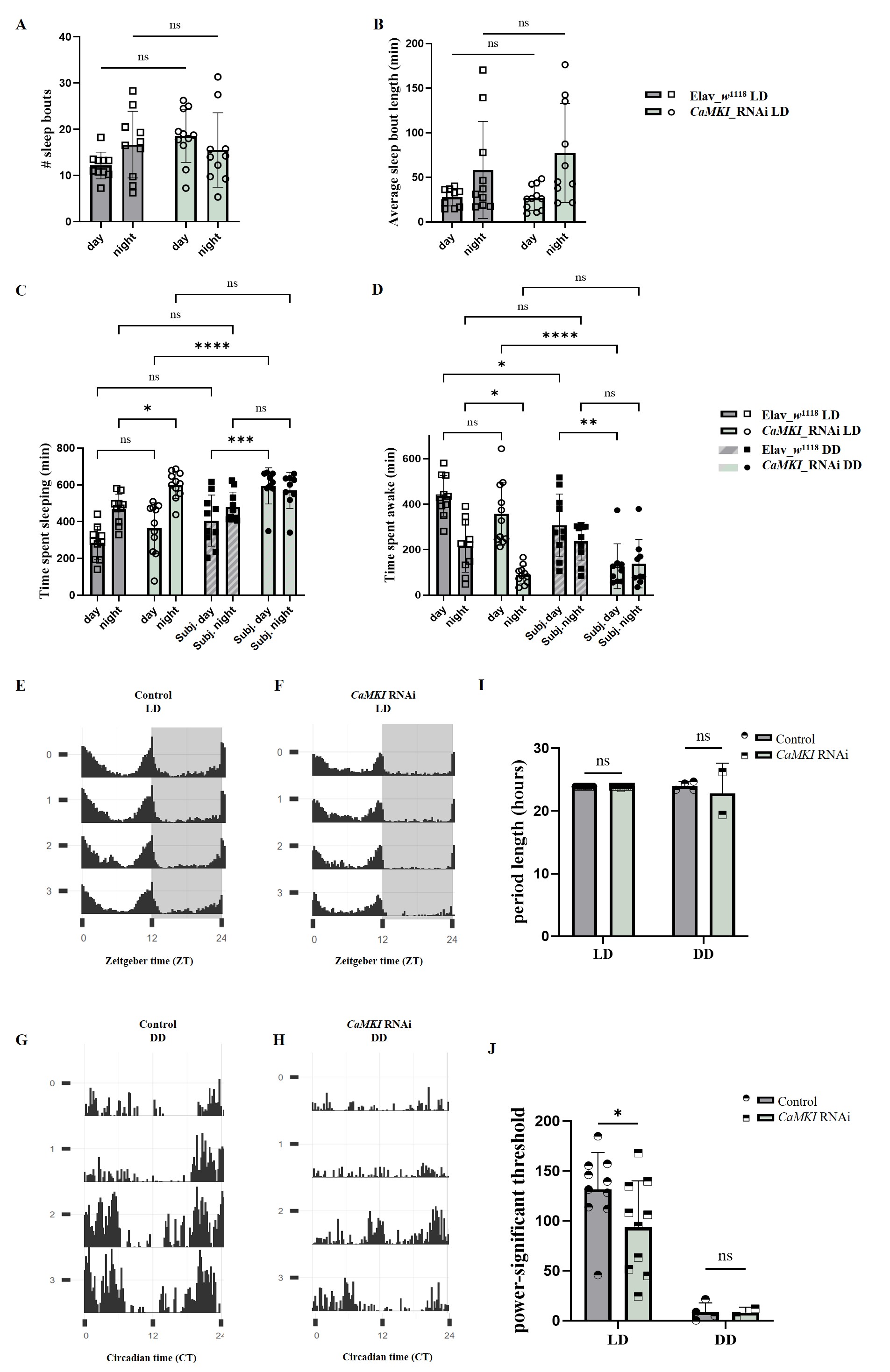

Supplement: Supplementary file 1 [file biology-14-01228-s001.zip › Figure S1. S1600.jpg]

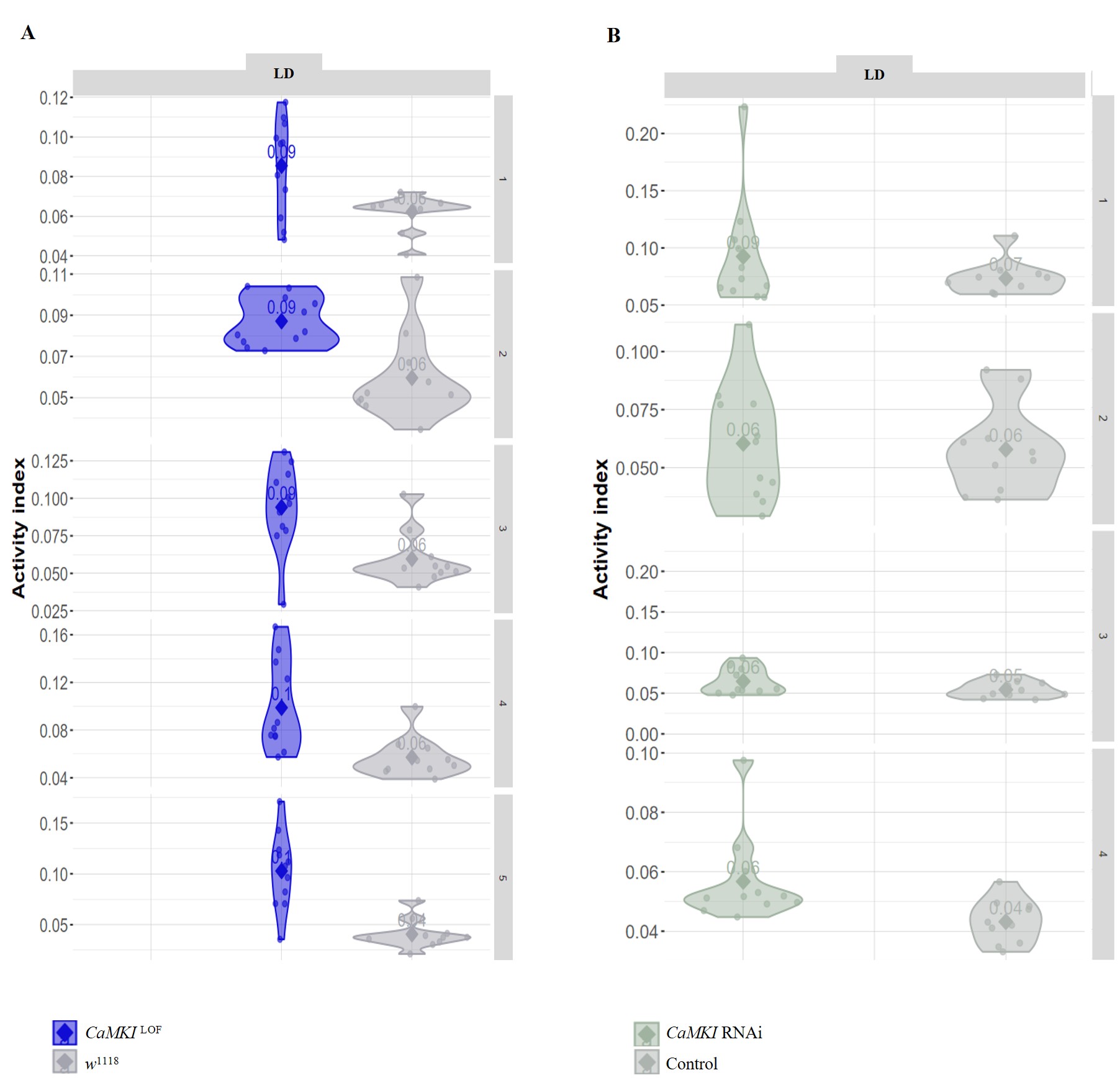

Supplement: Supplementary file 1 [file biology-14-01228-s001.zip › Figure S2. S2600.jpg]
